# Knowledge and awareness of sickle cell disease: a cross sectional study amongst unmarried adults in Nigeria’s capital city

**DOI:** 10.1007/s12687-022-00607-x

**Published:** 2022-09-28

**Authors:** Obi Peter Adigwe

**Affiliations:** National Institute for Pharmaceutical Research and Development, Federal Capital Territory, Plot 942, Cadastral Zone C16, Idu Industrial District, P.M.B. 21, Garki, Abuja, Nigeria

**Keywords:** Haemoglobin, Health, Anaemia, Blood, Sickle cell disease

## Abstract

Sickle cell disease is a genetic disorder characterised by the tendency of haemoglobin to polymerise and deform red blood cells to a sickle or crescent shape; this consequently results in vaso-occlusive condition. A better knowledge and awareness about sickle cell disease amongst the population can help reduce its prevalence. This study aimed at assessing awareness and knowledge of unmarried adults in Nigeria’s capital. A cross sectional survey was undertaken amongst unmarried individuals residing in the Federal Capital Territory. Questionnaires were administered to participants using convenience sampling strategy. Data were analysed using Statistical Package for Social Sciences version 25. Descriptive and inferential statistical analyses were carried out. A total of 1423 questionnaires were completed and returned, response rate was 83.71%, male participants were in the majority as indicated by 52% of the sample, and the dominant age group was 21 to 30 years (47.90%). Almost all the study participants (92.50%), have heard about sickle cell disease. Knowledge about sickle cell disease was average, as mean score for all the participants was 9.01 ± 3.18, with a range of 0 to 17. Some misconceptions were observed, for instance some participants believed that bacterial or viral infections could cause sickle cell disease. Male participants had a higher knowledge score compared to females (*p* < 0.001), and older participants were more knowledgeable about sickle cell disease (*p* < 0.001). This study identified that knowledge gaps exist about sickle cell disease. Emergent findings can underpin government, policymakers’ and stakeholders’ contextual strategies to prevent sickle cell through public health enlightenment and other relevant means.

## Introduction

Sickle cell disease is an autosomal recessive genetic red blood cell disorder characterised by sickling, a condition in which red blood cells become crescent or sickle shape as against the normal disc-shaped that is flexible enough to move easily through the blood vessels. The sickle shape occurs due to a mutation in the haemoglobin gene, and this decreases the cells’ flexibility. When the abnormal haemoglobin S appears in homozygous form (Hb SS), it is referred to as sickle cell anaemia, and it can also affect individuals in combination with other abnormal haemoglobins such as sickle cell haemoglobin C disease (Hb SC) and sickle cell β-thalassaemia (Hb Sβ-Thal) (Okpala 2004). They all possess a common tendency to turn red blood cells into a crescent shape under certain conditions, thereby resulting to several effects such as anaemia, jaundice, recurrent bone pain, and gradual deterioration of tissue and organ function (Nur et al. 2011). A person that receives one defective gene from both parents develops the disease, whilst an individual that receives one defective and one healthy gene remains healthy but can pass the disease, and such persons are referred to as carriers.

According to World Health Organisation, sickle cell trait is known to be widespread, with highest prevalence occurring in Africa, as it ranges between 10 and 40% in some areas (Oludare and Ogili 2013). In Nigeria, there is an estimate carrier prevalence of 25%, and 2–3% of National population suffers from the disease (Afolayan and Jolayemi 2011). Up to 20 per 1000 births are estimated to be affected with the disease, thereby resulting in about 150,000 children being born with sickle cell disease annually in the country (Anie et al. 2010). Thus, Nigeria is reported to have the largest population of persons affected with this condition (Ugwu 2016). Sickle cell disease is reported to have a remarkable public health implication for Africa, as it contributes about 5% to under-five deaths in the continent and up to 16% in West Africa (WHO 2006).

Despite the high prevalence of sickle cell disorder in Nigeria, available evidence suggests that the level of awareness and knowledge about the disease still remains low amongst youths of marriageable age. A study undertaken amongst fresh graduates in Ilorin, Nigeria reported poor knowledge about the disease, as more than half of participants in that study lacked understanding of the disease condition (Adewuyi 2000). A similar finding was also reported in Benin City where more than half of the participants indicated that they do not know their genotype, and only few respondents in that study had good knowledge about sickle cell disease (Bazuaye and Olayemi, 2009). Another study undertaken in Jos reported that a quarter of the participants wrongly believed that sickle cell disease was caused by evil spirits (Olarewaju et al. 2013). In Nigeria, people can easily get tested for their genotype on request in public and private healthcare facilities or laboratories with an average cost of 2,000 Naira, which is approximately 5 USD. Also, some religion organisations make genotype testing compulsory for couples as a prerequisite to the marriage ceremony (Ezugwu et al. 2019).

Wide knowledge about sickle cell disease is considered an important strategy in its prevention, as this can provide an opportunity for people to take informed decisions concerning marriage and procreation. Consequently, assessing the of knowledge of unmarried adults about sickle cell disease can help to develop appropriate public health programmes to increase awareness and knowledge about the condition. Several studies have reported knowledge and awareness about sickle cell disease amongst youths in Nigeria (Adewuyi 2000; Bazuaye and Olayemi 2009; Olarewaju et al. 2013; Adewoyin et al. 2015), but there is however paucity of information on knowledge and awareness about the condition amongst unmarried adults in the Federal Capital Territory. Few studies undertaken in this setting were focused on students (Owolabi et al. 2011; Yalma and Awodiji 2021); no robust study targeting the entire unmarried population has been undertaken in this area. This study therefore aimed to assess the level of awareness and knowledge of sickle cell disease amongst unmarried adults residing in the Federal Capital Territory, which is the capital of Nigeria.

## Methods

A cross-sectional study was undertaken in the Federal Capital Territory, Nigeria. Data collection commenced in August and ended in October 2021. The data collection tool was adapted from similar studies (Ugwu 2016; Boadu and Addoah 2018), with slight modifications. Face and content validations were carried out on the questionnaire using an expert panel comprising faculty members with research experience in the area of sickle cell disease. The data collection instrument was pretested by administering it to initial cohort of 40 participants randomly selected. The feedback received did not warrant any major change. Questionnaires were then administered physically using convenience sampling strategy (Etikan et al. 2016). The purpose of the study was explained to the respondents prior to questionnaire administration. Strategic locations which include motor parks, worship centres, and corporate offices were visited to recruit participants for the study. Inclusion criterial were unmarried adults and must be a resident in Federal Capital Territory. Participants were asked to indicate their marriage status prior to questionnaire administration, and those that were married were excluded from the study.

Ethical approval was obtained from Federal Capital Territory Health Research Ethics Committee before the commencement of data collection. Participation in the study was voluntary as informed consent was sought prior to the administration of questionnaires. Confidentially and anonymity were strictly maintained throughout the data collection process, as all information that could link participants to their responses were not included in the research instrument.

Completed questionnaires were retrieved from respondents and entered into Statistical Package for Social Sciences version 25. Descriptive statistical methods were used to summarise data on socio-demographic characteristics, awareness, and knowledge about sickle cell disease. Data were presented as frequencies (*n*) and percentages (%) for categorical variables. Knowledge about sickle cell disease was assessed by answering 17 multiple choice questions comprising correct and incorrect statements. The calculation of a total cumulative knowledge score for each participant was carried out. Questions were assigned one point for correct response and zero point for unanswered question or incorrect answer. The maximum score each participant can get was 17, and the minimum was 0.

Independent *t* test and analysis of variance (ANOVA) tests were employed to determine the relationships between mean knowledge score and socio-demographic variables. In the case of a significant ANOVA test, post hoc analysis (LSD) was performed for multiple comparisons between each two categories. A *p*-value of 0.05 or less was considered the threshold for statistical significance.

## Results

### Demography

Of the 1700 questionnaires administered, a total of 1423 copies were completed and returned, giving a response rate of 83.71%. Male participants represented 52.00% of the sample which was slightly higher than females. Close to half of the respondents (47.90%) were between 21 and 30 years, whilst those of 20 years and below represented a third of the sample (31.60%). Further details about socio-demographic characteristics are presented in Table 1.

**Table 1:** Socio-demographic characteristics

Variable	Frequency (%)
Gender	
Male	740 (52.00)
Female	683 (48.00)
Age	
≤ 20	445 (31.60)
21–30	674 (47.90)
31 and above	289 (20.50)
Highest level of education	
Primary education	47 (3.50)
Secondary education	372 (27.80)
Ordinary national diploma	339 (25.40)
First degree/HND	518 (36.40)
Postgraduate	61 (4.60)
Do you know your genotype	
No	200 (14.40)
Yes	1189 (83.60)

Abbreviation: *HND*, higher national diploma

### Knowledge and awareness about sickle cell disease

Findings from this study revealed that a strong majority of the participants (92.50%) were aware of sickle cell disease, whilst only a small proportion (7.50%) indicated that they have never heard of the disease.

The knowledge score ranged from 0 to 17, with a total mean of 9.01 ± 3.18. The majority of the participants (88.00%) indicated correctly that sickle cell disease is a group of blood disorder inherited from a person’s parents. Only about 40.90% of the participants disagreed that some bacteria in the blood can cause sickle cell disease, whilst others were either not sure of the answer or answered wrongly by indicating “true” as their response. More than two-thirds (70.90%) of the respondents answered correctly that a person with Hb SS genotype is said to have sickle cell trait or referred to as a carrier, whilst only slightly above a quarter of the participants answered correctly that someone with sickle cell trait will not possess symptoms of the disease. The majority of the participants (70.8%) indicated that genetic counselling was a method of controlling sickle cell disease. Other relevant details on knowledge about sickle cell disease are presented Table 2.

**Table 2:** Knowledge about sickle cell disease (those that answered correctly)

SN	Statement	Frequency (%)
1	Sickle cell disease is a group of blood disorders typically inherited from a person’s parents	1252 (88.00)
2	Some bacteria in the blood can cause sickle cell disease	582 (40.90)
3	A person with AS genotype is said to have sickle cell trait or referred to as carrier	1009 (70.90)
4	A person with sickle cell trait usually have symptoms of sickle cell disease	390 (27.40)
5	Diagnosis of sickle cell disease is possible during pregnancy	801 (56.30)
6	Some viral infection in the blood can also cause sickle cell disease	640 (45.00)
7	A large proportion of sickle cell disease occurs in sub-Saharan Africa	452 (31.80)
8	People with sickle cell disease are less vulnerable to malaria	667 (46.90)
9	Only one of the parents contributes to sickle cell disease transmission to offspring	807 (56.70)
10	New born screening programme is an appropriate time to test for sickle cell disorder	666 (46.80)
11	Genetic counselling is a method of controlling sickle cell disease	1008 (70.80)
12	Prenatal diagnosis can help to prevent sickle cell disease	827 (58.10)
13	Pain is one of the commonest presenting symptoms of sickle cell disease	768 (54.00)
14	More than 80% of persons suffering from sickle cell disease will die before the age of 20 years	651 (45.70)
15	Sickle cell disorder is a communicable disease	844 (59.30)
16	Treatment exists for sickle cell disease	685 (48.10)
17	Sickle cell disease affects red blood cell	527 (37.00)

### Sources of information about sickle cell disease

**Fig. 1:**
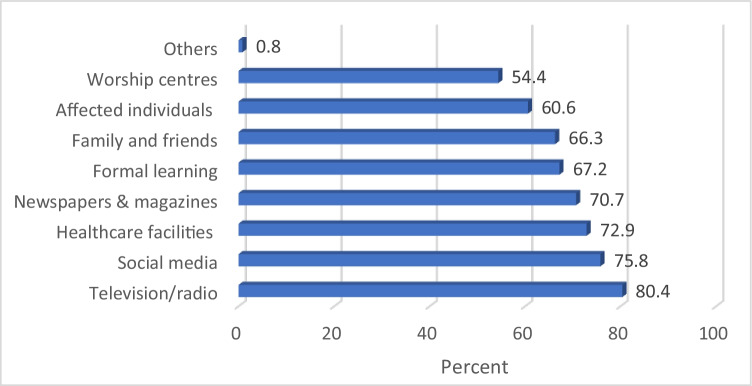
Overview of sources of information about sickle cell disease

The major sources of information about sickle cell disease were television/radio (80.40%), social media (75.80%), healthcare facilities (72.90%), followed by newspapers and magazines. Figure 1 below gives an overview of sources of information about sickle cell disease.

The finding in Fig. 1 shows that electronic media represented the common source of information about sickle cell disease. Also, the internet had also played key role regarding knowledge about the disease as three quarters of the study participants got their information from social media.

### Relationship between demographic characteristics and knowledge about sickle cell disease

Inferential statistical analyses undertaken shows there was a significant relationship between the participants socio-demographic characteristics and their knowledge about sickle cell disease. Male respondents had a higher knowledge score than female participants, and this finding was statistically significant (*p* < 0.001). Older adults had better knowledge about sickle cell disease (*p* < 0.001). Those who knew their genotype also had a better knowledge regarding the disease (*p* = 0.004). Other details on association between socio-demographic variables and knowledge are presented in Table 3.

**Table 3:** Relationship between knowledge score and demographic factors

Variable	Category	Mean ± SD	Test of significance (*p*)
Gender			*t* = − 3.563 (< 0.001)
Male		8.72 ± 3.20	
Female		9.32 ± 3.14	
Age			*F* = 9.964 (< 0.001)
≤ 20	a	8.57 ± 3.15	a vs b (0.021); a vs c (< 0.001); b vs c (0.005)
21–30	b	9.01 ± 2.96	
31 and above	c	9.63 ± 3.62	
Highest level of education			*F* = 33.788 (< 0.001)
Primary education	a	5.36 ± 3.44	a vs b (< 0.001); a vs c (< 0.001); a vs d (< 0.001); a vs e (< 0.001); b vs c (< 0.001); b vs c (< 0.001); b vs d (< 0.001); b vs e (0.144); c vs d (0.001) c vs e (0.389); d vs e (0.011)
Secondary education	b	8.19 ± 3.08	
Ordinary national diploma	c	9.17 ± 3.11	
First degree/HND	d	9.85 ± 2.88	
Postgraduate	e	8.80 ± 3.49	
Knows genotype			*t* = − 5.235 (0.004)
Yes		9.22 ± 3.06	
No		7.97 ± 3.59	

*F*, analysis of variance (ANOVA) test; *t*, independent *t* test.

## Discussion

This study was undertaken to assess awareness and knowledge of unmarried adults resident in the Federal Capital Territory about sickle cell disease. Findings from this study revealed almost all the participants were aware of sickle cell disease, and this result is comparable with similar studies carried out in other parts of Nigeria (Adewoyin et al. 2015; Ugwu 2016). The reason behind this, may be the high prevalence of sickle cell disease in the country (Afolayan and Jolayemi 2011).

Despite the high level of awareness recorded in this study, the majority of respondents lacked adequate knowledge about the disease. In general, the mean knowledge score for the study participants was just a little above average, suggesting lack of comprehensive knowledge about the disease. This finding is also similar to previous research (Adeodu et al. 2000; Moronkola and Fadairo 2006; Alao and Araoye 2009), but is also at variance with other studies that reported good knowledge about sickle cell disease in some parts of Nigeria (Adeyemo et al. 2007; Gbeneol et al. 2015; Omuemu et al. 2013). As an urban city which doubles as the capital of Nigeria, one expects that the population in such setting would have good knowledge about the disease. Findings from this study therefore raises concerns about the less than optimal knowledge and awareness about the disease.

Although the majority of the study participants knew that sickle cell disease is a blood disorder, there were however some misconceptions, as a significant proportion linked bacterial and viral infections with the disease. Other misconceptions that emerged in the study include the notion that sickle cell disease is a communicable disease, and more than 80% of those affected with the condition would die before the age of 20 years. These findings mirror previous study undertaken in Benin City where participants expressed various forms of misconceptions concerning the disease (Zounon et al. 2012). Although sickle cell disease patients have between 20 to 30 years shorter life expectancy than the general population (Houwing et al. 2021), however, with comprehensive healthcare programme, almost all infants are expected to survive into adulthood (Couque et al. 2016; Gardner et al. 2016). Close to three quarters of the study participants answered correctly that a person with haemoglobin phenotype AS is referred to as carrier, this was expected considering the fact that the majority participants knew their genotype. However, a considerable proportion of the respondents felt that someone with sickle cell trait may also exhibit some symptoms of sickle cell disorder. This finding is an indication of lack of adequate knowledge about sickle cell disease. The majority of participants had knowledge about genetic counselling as a means of preventing sickle cell disease, and this is in line with earlier finding by Adeyemo et al. (2007) in a study carried out amongst students in the University of Lagos.

In this study, participants indicated television/radio as the major source of information about sickle cell disease; this was closely followed by social media, healthcare facilities, and newspapers and magazines. Only about two-thirds of the participants indicated formal learning as source of information, and this was in contrast with other studies which had formal learning as the common source of information about sickle cell disease (Boyd et al. 2005; Al-Farsi et al. 2014; Boadu and Addoah, 2018).

Furthermore, inferential statistical analyses undertaken showed that male participants were more knowledgeable about sickle cell disease compared to female respondents; the reason behind this is however not clear; further studies can be undertaken in order to unravel the rationale behind this emergent, yet significant correlation. Findings from this study revealed that both age and educational qualifications had a significant associations with knowledge of the participants about sickle cell disease. With respect to age, the older participants were more knowledgeable than the younger participants, as knowledge was observed to increase across the various age categories. Whilst for educational level, those who had only primary education had poor knowledge about the disease, and this finding was consistent with that reported by Oludare and Ogili (2013). This implies that more sensitisation needs to be targeted towards this group. There is also a need for government to develop contextual strategies that would include awareness improvement from primary education stage. An increase in mean knowledge score was observed as level of education increased from primary school level to first degree. Surprisingly, respondents who were educated up to postgraduate level had a lower mean knowledge score compared to those with first degree. This may however be due to the fact that few postgraduate respondents participated in the study. In addition, findings from this study also showed that participants who knew their genotype had better knowledge about the disease. This may be attributed to the fact that this group of persons may have received some form of counselling at the point of testing which consequently improved their knowledge about the disease.

In the study design, a robust sampling strategy ensured inclusion of a significant number of participants from a population of unmarried adults in the Nigerian Federal Capital Territory. Whilst this underpinned the emergence of novel findings, there may be limitations to the external validity of the study. A National study will therefore be required to address some of the study gaps as well as significantly improve the generalisability of the outcomes.

## Conclusion

This study has revealed important insights about knowledge and awareness about sickle cell disease amongst unmarried adults residing in Nigeria’s capital city. Findings from this research can help the government and policymakers in developing a robust framework to help address knowledge gaps in the study area.

Although most of the participants in this study were aware of the existence of sickle cell disease, they however lacked adequate knowledge regarding critical aspects of the disease. Male participants had higher knowledge score compared to females, and older participants had a better knowledge about the disease compared to younger respondents. Level of education also had influence on knowledge about the disease.

Given the relative prevalence and burden of sickle cell disease in Nigeria, it is critical for government, policymakers, and relevant stakeholders to collectively develop comprehensive and contextual strategies that can underpin effective dissemination of knowledge amongst different population groups in the country.

Although this study has provided some insights into knowledge and awareness of young adults in Federal Capital Territory regarding sickle cell disease, a better understanding from this group’s perspective emerged as invaluable in developing policies that can help increase knowledge about the disease amongst young people. Therefore, further research needs to be undertaken to build on these novel findings and broaden the evidence base.

## Data Availability

The datasets generated and analysed during this study are available on request.
